# Adenylyl cyclase subtype 1 is essential for late-phase long term potentiation and spatial propagation of synaptic responses in the anterior cingulate cortex of adult mice

**DOI:** 10.1186/1744-8069-10-65

**Published:** 2014-10-10

**Authors:** Tao Chen, Gerile O’Den, Qian Song, Kohei Koga, Ming-Ming Zhang, Min Zhuo

**Affiliations:** Center for Neuron and Disease, Frontier Institutes of Life Science and of Science and Technology, Xi’an Jiaotong University, Xi’an, 710049 China; Department of Physiology, Faculty of Medicine, University of Toronto, 1 King’s College Circle, Toronto, Ontario M5S 1A8 Canada; Department of Anatomy & K.K. Leung Brain Research Center, Fourth Military Medical University, Xi’an, ShaanXi 710032 China

**Keywords:** Adenylyl cyclase 1, Gabapentin, Anterior cingulate cortex, LTP, Chronic pain

## Abstract

Long-term potentiation (LTP) is a key cellular mechanism for pathological pain in the central nervous system. LTP contains at least two different phases: early-phase LTP (E-LTP) and late-phase LTP (L-LTP). Among several major cortical areas, the anterior cingulate cortex (ACC) is a critical brain region for pain perception and its related emotional changes. Periphery tissue or nerve injuries cause LTP of excitatory synaptic transmission in the ACC. Our previous studies have demonstrated that genetic deletion of calcium-stimulated adenylyl cyclase 1 (AC1) or pharmacological application of a selective AC1 inhibitor NB001 blocked E-LTP in the ACC. However, the effect of AC1 on L-LTP, which requires new protein synthesis and is important for the process of chronic pain, has not been investigated. Here we tested the effects of NB001 on the ACC L-LTP and found that bath application of NB001 (0.1 μM) totally blocked the induction of L-LTP and recruitment of cortical circuitry without affecting basal excitatory transmission. In contrast, gabapentin, a widely used analgesic drug for neuropathic pain, did not block the induction of L-LTP and circuitry recruitment even at a high concentration (100 μM). Gabapentin non-selectively decreased basal synaptic transmission. Our results provide strong evidence that the selective AC1 inhibitor NB001 can be used to inhibit pain-related cortical L-LTP without affecting basal synaptic transmission. It also provides basic mechanisms for possible side effects of gabapentin in the central nervous system and its ineffectiveness in some patients with neuropathic pain.

## Introduction

Long term potentiation (LTP) of synaptic transmission is believed to be a key cellular mechanism for learning and storing sensory information in the brain [1–5]. For pathological pain, LTP is triggered by peripheral injury both in the spinal cord [6] and in sensory-related cortical areas [4, 5, 7]. In learning-related hippocampus, LTP contains at least two different phases: early-phase LTP (E-LTP), which does not need new protein synthesis or transcription, and late phase LTP (L-LTP), which is transcription and translation-dependent [2, 8–10]. Similarly, in sensory and emotion-related cortical areas such as the insular cortex (IC) and anterior cingulate cortex (ACC), both E-LTP and L-LTP have been recently reported in adult mice [11–14].

Among several cortical areas, cumulative evidences from animal and human studies suggest that the ACC is important for chronic pain. The ACC plays important roles not only for the perception and regulation of pain, but also for pain related emotional changes [5, 15]. Our previous results showed that LTP of excitatory synaptic transmission was observed in the ACC of mice with chronic pain [16–20]. Supporting the role of ACC LTP in chronic pain, inhibiting the induction or expression of LTP in the ACC produces significant analgesic effects in animal models of chronic pain [16, 17, 21, 22]. Therefore, proteins and ion channels in the ACC involved in the induction and expression of LTP can serve as potential drug targets for treating chronic pain [7, 23, 24]. Among several candidates, the N-methyl-D-aspartic acid (NMDA) receptor and its downstream signal molecules are important for synaptic potentiation [1, 25, 26]. Our previous studies have found that calcium-stimulated adenylyl cyclase 1 (AC1), a main downstream signal protein of NMDA receptor, is essential for the induction of E-LTP in the ACC [17, 19, 21, 24, 27]. Application of a selective AC1 inhibitor NB001 produced powerful analgesic effects in different animal models of chronic pain, including neuropathic pain, inflammatory, muscle pain and visceral pain [21, 28, 29]. While E-LTP is likely important in the early onset of chronic pain, L-LTP is critical for chronic pain development [11, 16, 30]. However, the evidence for the role of AC1 in ACC L-LTP is still lacking.

In the present study, we used a 64-channel multi-electrode array recording system (MED64) to record L-LTP in the ACC, and tested the effect of NB001 on its properties. To compare the effect of NB001 with other key analgesic compounds, we also examined the effect of gabapentin on the ACC L-LTP. Gabapentin is a widely used drug for treating neuropathic pain [31, 32]. However, gabapentin’s potential effect on cortical LTP has not been investigated. We found that application of NB001 completely blocked the induction of L-LTP and the propagation of the evoked potentials without affecting the basal excitatory transmission. In contrast, gabapentin did not block the induction of ACC LTP and propagation of evoked potentials but decreased the basal excitatory synaptic transmission.

## Results

### The network L-LTP within the ACC by multi-channel recording

**Figure 1:**
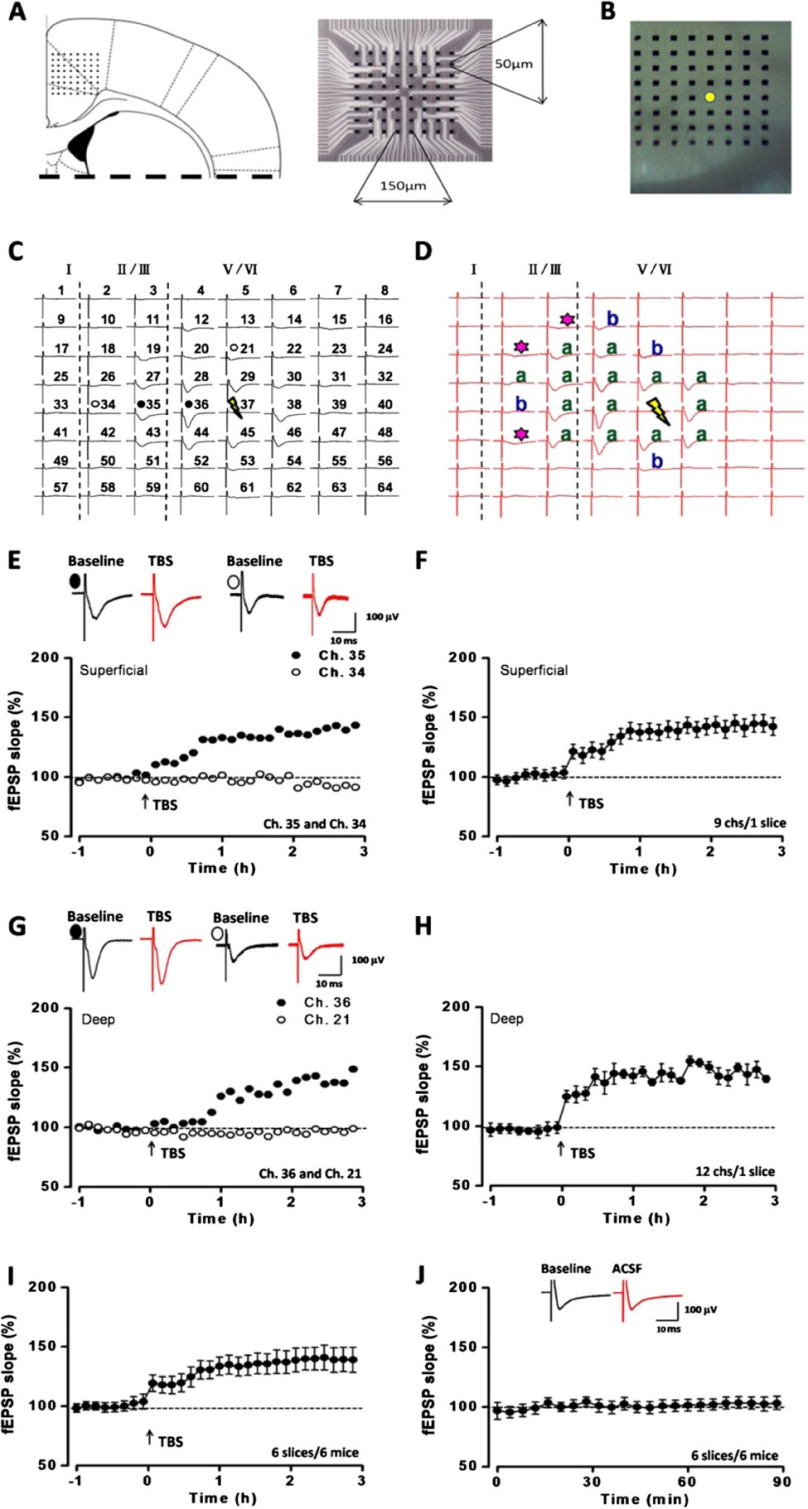
**Spatial distribution of network synaptic transmission in the ACC slice on a multi-channel array probe. (A)** Schematic diagram of location of an ACC slice on the MED-64 probe (*left*) and the scale of the electrodes (*right*). **(B)** Light microscopy photograph showing the relative location of ACC slice and MED-64 probe. The yellow circle is the selected stimulated site. **(C-D)** Two mapped figures showed the evoked potentials in all active channels in 1 slice 0.5 h before (black) and 3 h after TBS (yellow lightning bolt). Channels with L-LTP and no L-LTP were marked as “a” (green) and “b” (blue), and the TBS-recruited synaptic responses were marked as asterisks, respectively. Vertical lines indicated the layers of ACC slices. **(E)** The sample traces and the plotted slope results of one channel showing L-LTP (Ch. 35) and the other without showing L-LTP (Ch. 34) of the fEPSP from one slice in the superficial layer. **(F)** The plotted slope showed the L-LTP of all 6 channels in the superficial layer. **(G)** The sample traces and the plotted slope results of one channel showing L-LTP (Ch. 36) and the other without showing L-LTP (Ch. 21) of the fEPSP from the same slice in the deep layer. **(H)** The plotted slope showed the L-LTP of all 12 channels in the deep layer. **(I)** The summarized L-LTP of the fEPSP slopes of 6 slices/6 mice. **(J)** The summarized fEPSP slopes of 6 slices/6 mice showed that, if no TBS induction was applied, the basal synaptic transmission kept stable.

A MED64 recording system has been recently used to map synaptic responses from different layers of adult mouse ACC [11, 16, 33]. In the present study, we first examined the network L-LTP in the ACC of adult C57 mice. The location of the 8x8 array MED64 probe electrodes within the ACC slice is shown in Figure 1A and B. One channel (Figure 1B, yellow circle) that located on the deep layer V of the ACC was chosen as the stimulation site and field excitatory post-synaptic potentials (fEPSPs) recorded from the other channels around the stimulation site were recorded. Figure 1C showed an example of such experiments and fEPSPs were recorded from 18 channels in both deep (V/VI) and superficial layers (II/III) of the ACC. After one hour of stable baseline recording, theta burst stimulation (TBS) was applied to induce L-LTP. We found that TBS induced stable L-LTP that lasted for 3 h in most of the active channels (Figure 1D). The fEPSPs slopes and the total number of activated channels including those with or without L-LTP in both superficial and deep layers were then analyzed. Figure 1E indicates the slope of two channels both located in the superficial layers: Ch. 35 showed a long-lasting LTP (141.9% of baseline at 3 h after TBS) but Ch. 34 did not undergo any potentiation (94.6% of baseline at 3 h after TBS). The averaged data from 6 channels in superficial layers of the sample slice was plotted in Figure 1F (LTP: 146.3 ± 9.2% of baseline at 3 h after TBS). In deep layers, similar results were observed and the slope of two channels (Ch. 34 and 36) were plotted in Figure 1G: Ch. 36 showed a long-lasting LTP (141.2% of baseline at 3 h after TBS) but Ch. 34 did not show any potentiation (98.3% of baseline at 3 h after TBS). The averaged data from 12 channels in deep layers of the same slice was plotted in Figure 1H (LTP: 145.3 ± 6.2% of baseline at 3 h after TBS). L-LTP was similar within both the superficial and deep layer. We found the fEPSP from 77.8% of all active channels were significantly potentiated (77 from 99 active channels; 6 slices/6 mice). The mean percentage of potentiation was 155.2 ± 4.4% in the 77 channels. In other 22 channels, no obvious potentiation was detected. The overall changes of all 99 channels was 140.4 ± 10.3% of the baseline at 3 h after TBS (*P* < 0.001, paired t-test, Figure 1I).

### NB001 blocked the network L-LTP without affecting the basal transmission

**Figure 2:**
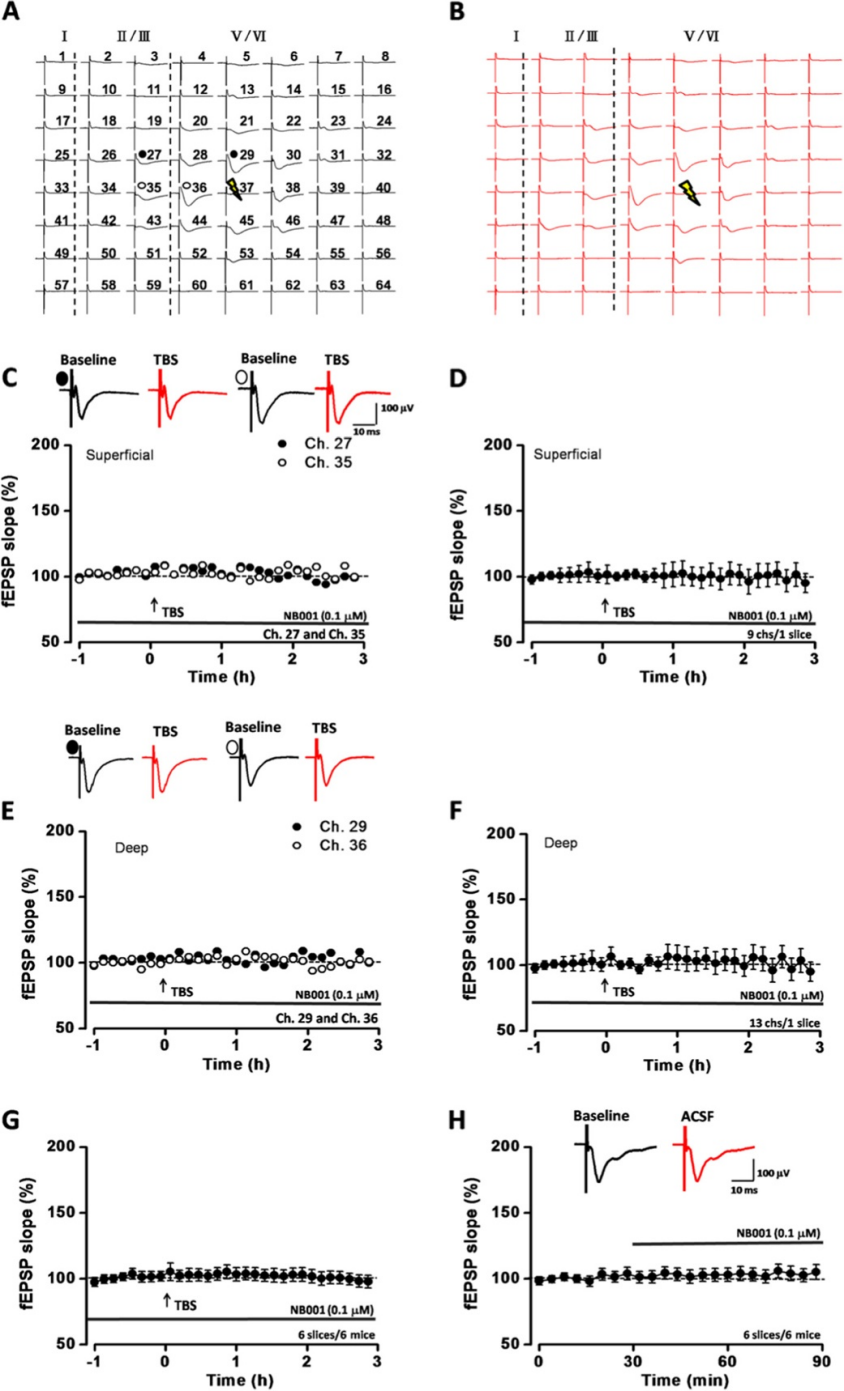
**Basic synaptic transmission and long-term potentiation in the ACC with NB001 application. (A-B)** Two mapped figures showed the LTP of the fEPSP slope was blocked in the presence of NB001 (0.1 μM). The baseline responses **(A)** were not potentiated 3 h after TBS applied on one channel (yellow lightning bolt) **(B)**. **(C)** The sample traces and the plotted slope results of two channels (Ch. 27 and Ch. 35) in superficial layer from one slice showed the potentiation of the fEPSP was blocked with NB001 application. **(D)** The plotted slope from 9 channels in superficial layer was not potentiated with NB001 application. **(E)** The sample traces and the plotted slope results of two channels (Ch. 29 and Ch. 36) in deep layer from the same slice showed the potentiation of the fEPSP were both blocked with NB001 application. **(F)** The plotted slope from 13 channels in deep layer was not potentiated with NB001 application. **(G)** The summarized fEPSP slopes of 6 slices/6 mice showed that NB001 application blocked the L-LTP induction. **(H)** The summarized fEPSP slopes of 6 slices/6 mice showed that NB001 application did not change the basal synaptic transmission.

A previous study has shown that an AC1 inhibitor NB001 blocks E-LTP in the ACC [21]. Here we tested whether bath application of NB001 could induce the similar blocking effect on the network L-LTP in the ACC slices. In the presence of NB001 (0.1 μM), we found that L-LTP induction was totally blocked both in superficial and deep layers (Figure 2A-G). Figure 2C showed an example of two active channels (Ch. 27 and 35) in the superficial layers with NB001 application. After TBS, they all failed to undergo any potentiation (Ch. 27 was 102.0% and Ch. 35 was 98.3% of baseline at 3 h after TBS). In a total of 9 channels in the sample slice, TBS did not induce L-LTP (101.4 ± 6.3% of baseline at 3 h after TBS) in the superficial layers (Figure 2D). Similar results were found in deep layers (Figure 2E-F). In two randomly selected active channels (Ch. 29 and 36), no potentiation was observed (Ch. 29 was 103.1%; Ch. 36 was 100.2% of baseline at 3 h after TBS) (Figure 2E). TBS did not induce LTP in all 13 channels (106.2 ± 10.7% of baseline at 3 h after TBS) (Figure 2F). The mean slope of fEPSPs reached 98.5 ± 4.5% of the baseline at 3 h after TBS (n = 6 slices/6 mice; *P* = 0.085, paired t-test, Figure 2G). Meanwhile, bath application of NB001 did not change the basal synaptic transmission (103.0 ± 6.0% of the baseline after application for 1 h, n = 6 slices/6 mice, Figure 2H).

### Gabapentin reduced the basal transmission without affecting the L-LTP

**Figure 3:**
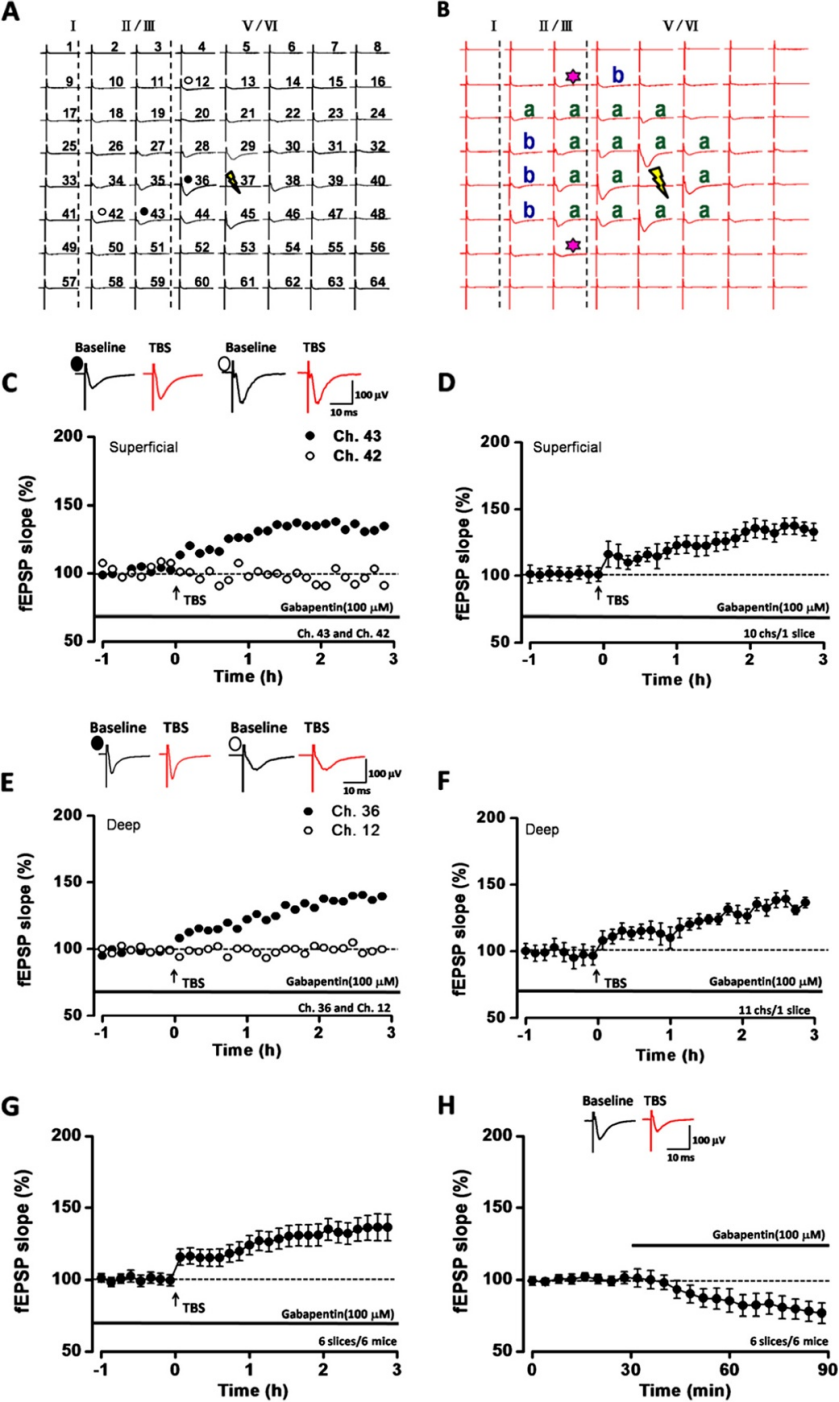
**Basic synaptic transmission and LTP in the ACC with gabapentin application. (A-B)** Two mapped figures showed the LTP of the fEPSP slope was induced in the presence of gabapentin (100 μM). The baseline responses **(A)** were potentiated 3 h after TBS applied on one channel (yellow lightning bolt) **(B)**. Channels with L-LTP and no L-LTP were marked as “a” (green) and “b” (blue), and the TBS-recruited synaptic responses were marked as asterisks. **(C)** The sample traces and the plotted slope results of one channel showing L-LTP (Ch. 43) and the other without showing L-LTP (Ch. 42) in the superficial layer with gabapentin application. **(D)** The plotted slope showed the L-LTP of the fEPSP of 8 channels in the superficial layer with gabapentin application. **(E)** The sample traces and the plotted slope results of one channel showing L-LTP (Ch. 36) and the other without showing L-LTP (Ch. 12) in the deep layer of the same slice with gabapentin application. **(F)** The plotted slope showed the L-LTP of 11 channels in the deep layer with gabapentin application. **(G)** The summarized fEPSP slopes of 6 slices/6 mice with gabapentin application. **(H)** The summarized fEPSP slopes of 6 slices/6 mice showed gabapentin decreased the basic synaptic transmission.

Gabapentin is an anti-epileptic agent and recommended as the first line agent for neuropathic pain treatment [34, 35]. It is believed that gabapentin binds to α2δ-1, a subunit of voltage gated calcium channel, to produce analgesia [36]. However, the effect of gabapentin on synaptic plasticity has not been reported yet. We then tested whether gabapentin affect the network L-LTP in the ACC. We found that even at a high concentration (100 μM), gabapentin had no effect on the L-LTP and most of the active channels in superficial and deep layers showed potentiation (Figure 3A-G). Ch. 42 and 43 were two randomly selected channels in the superficial layers. After TBS, Ch. 43 showed a long-lasting LTP (132.4% of baseline at 3 h after TBS) but Ch. 42 did not undergo any potentiation (95.9% of baseline at 3 h after TBS) (Figure 3C). The averaged data from 8 channels in superficial layers of the sample slice reached 137.6 ± 4.0% of baseline at 3 h after TBS) (Figure 3D). Similar results were observed in the deep layers. In Figure 3E, Ch. 36 showed a long-lasting LTP (137.8% of baseline at 3 h after TBS) but Ch. 12 did not have any potentiation (96.3% of baseline at 3 h after TBS). The averaged data from 11 channels in deep layers was 135.2 ± 10.0% of baseline at 3 h after TBS. In a total of 6 slices from 6 mice, the mean fEPSP slope of 97 active channels (76 channels with L-LTP, 78.4% of all activated channels) was 136.2 ± 9.0% of the baseline (*P <*0.001, paired t-test, Figure 3G). However, gabapentin application directly decreased the basal synaptic transmission (80.9 ± 7.4% of the baseline, after application for 1 h, n = 6 slices/6 mice; *P <*0.001, paired t-test; Figure 3H).

### Spatial distribution and recruitment of synaptic responses within the ACC network

**Figure 4:**
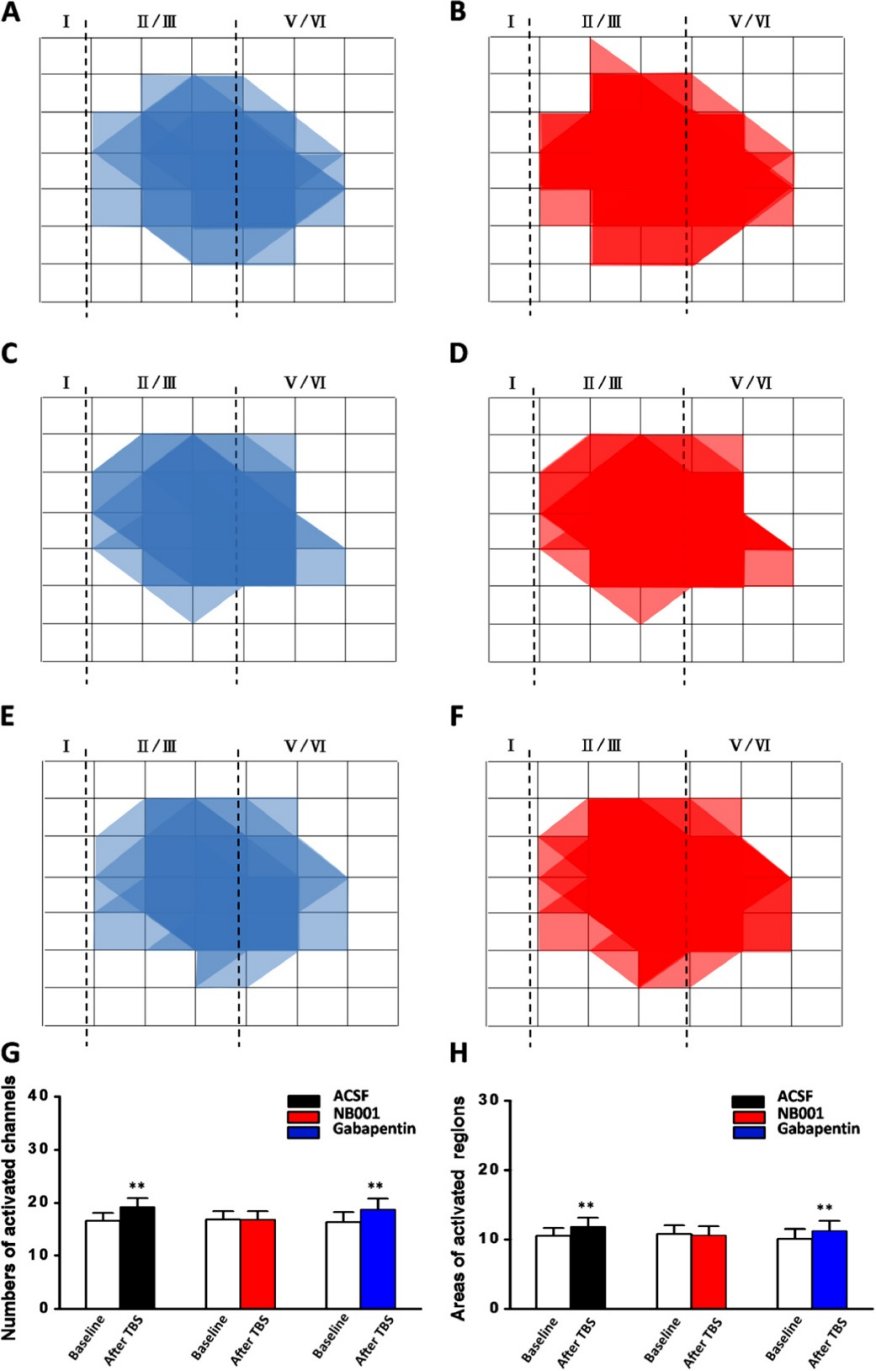
**Effect of NB001 and gabapentin on the spatial distribution induced by TBS. (A-B)** Polygonal diagrams of showed the baseline areas of the activated sites with fEPSPs (blue) and the enlarged areas after TBS (red) (n = 6 slices/6 mice). Overlapped blue or red regions indicated the high frequently activated areas. **(C-D)** Baseline areas of activated sites with fEPSPs (blue) was not enlarged after TBS (red) in the presence of NB001 (0.1 μM) (n = 6 slices/6 mice). **(E-F)** Baseline areas of activated sites with fEPSPs (blue) and the enlarged areas after TBS (red) with gabapentin (100 μM) application (n = 6 slices/6 mice). **(G-H)** Summarize number of active channels **(G)** and areas **(H)** that are activated before and after TBS induction.

Multi-channels recording provides a convenient way to study the cortical network L-LTP. We then mapped the spatial distribution of the active responses in the ACC before and after TBS by using the previously method [11, 12, 30, 33]. The distribution of all observed activated channels during the whole recording progress was displayed by a polygonal graph on a grid representing the channels (the blue lines represent the activated channels during the baseline and the red lines represent the activated channels after TBS). When we stimulated the local site in deep layer, the spread of active response was observed in both deep and superficial layers around the stimulation site. As described before, closer to the stimulation site, more channels could be activated. Moreover, the spreading leaned to the superficial layers (Figure 4A). During the baseline recording, 99 channels from 6 slices/6 mice were activated (26.2% of all 378 channels, 16.5 ± 1.5 per slice in average). However, sixteen channels that had no responses during baseline recording were recruited and showed responses at 3 h after TBS (4.2% of all 378 channels at 3h after TBS, Figure 4A-B, G). Similar to our previous results of Fmr1 WT mice [11], the recruited channels were located on the edge of the originally active area (Figures 1D, 4A).

We then tested the effect of NB001 and gabapentin on the spatial distribution and network recruitment in the ACC. We found that bath application of NB001 and gabapentin did not affect the number of activated channels (NB001:101 activated channels, 16.8 ± 1.5 per slice in average; gabapentin: 97 activated channels, 16.2 ± 1.8 per slice in average; n = 6 slices/6 mice in each group, *P* > 0.05 in comparison with the control group, unpaired t-test). However, NB001 but not gabapentin blocked the TBS induced channel recruitment (NB001, -1 recruited channels in total; gabapentin, 14 recruited channels in total, n = 6 slices/6 mice, Figure 4C-F, G). When standardizing the area of one grid as 1, the sum area of all channels in control slices was counted as 49. The average activated areas were 10.5 ± 1.1 and 11.8 ± 1.3 before and after TBS, whereas the increased area was 1.3 ± 0.0% per slice (*P* < 0.001, paired t-test, Figure 4H). Similarly, NB001 but not gabapentin blocked the increasing areas 3 h after TBS (NB001:10.8 ± 1.3 and 10.6 ± 1.4 before and after TBS, *P* = 0.175, paired t-test; gabapentin: 10.1 ± 1.4 and 11.3 ± 1.4 before and after TBS, *P* < 0.001, paired t-test. Figure 4H).

**Figure 5:**
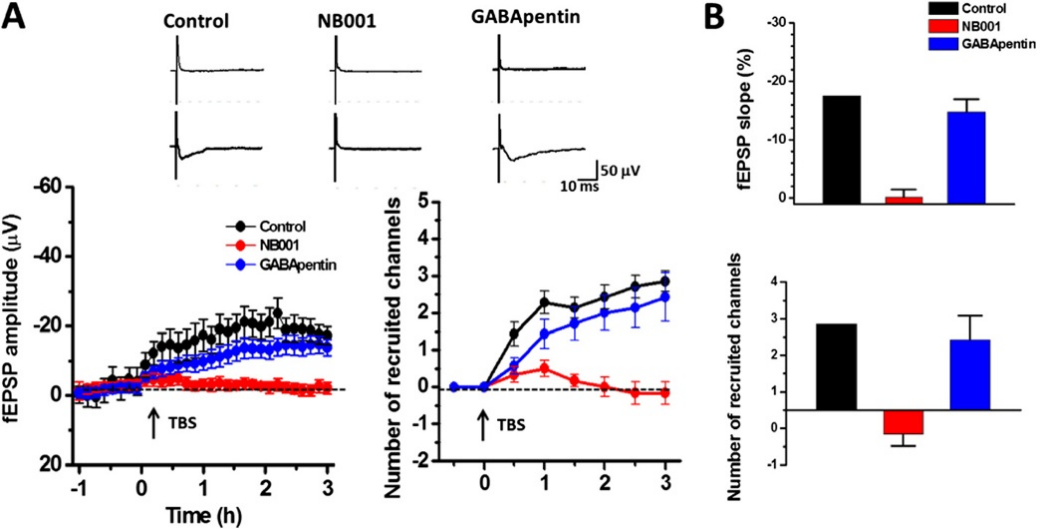
**The time course of recruited responses induced by TBS. (A)** Superimposed samples and summarized results showing the temporal changes of the EPSP amplitude and number of recruited responses. **(B)** The mean fEPSP slope and number of recruited channels are plotted.

The time course of the recruitment changes in control group were shown in Figure 5. The number of recruited channels gradually increased across the extended time scale after TBS induction and finally reached 2.7 ± 0.2 per slice at 3 h after induction. The averaged amplitude of the recruited responses also gradually potentiated and finally reached as large as 16.7 ± 2.3 μV. NB001 but not gabapentin blocked the TBS induced channel recruitment (NB001: -0.2 ± 0.2 recruited channels per slice at 3 h after TBS, *P* = 0.363, paired t-test; gabapentin: 2.3 ± 0.2 recruited channels per slice at 3 h after TBS, *P* < 0.001, paired t-test). The averaged amplitude of the recruited responses finally reached as 1.9 ± 0.3 μV or 14.2 ± 0.2 μV in the presence of NB001 or gabapentin.

### Effect of NB001 and gabapentin on E-LTP: whole-cell patch-clamp recording

**Figure 6:**
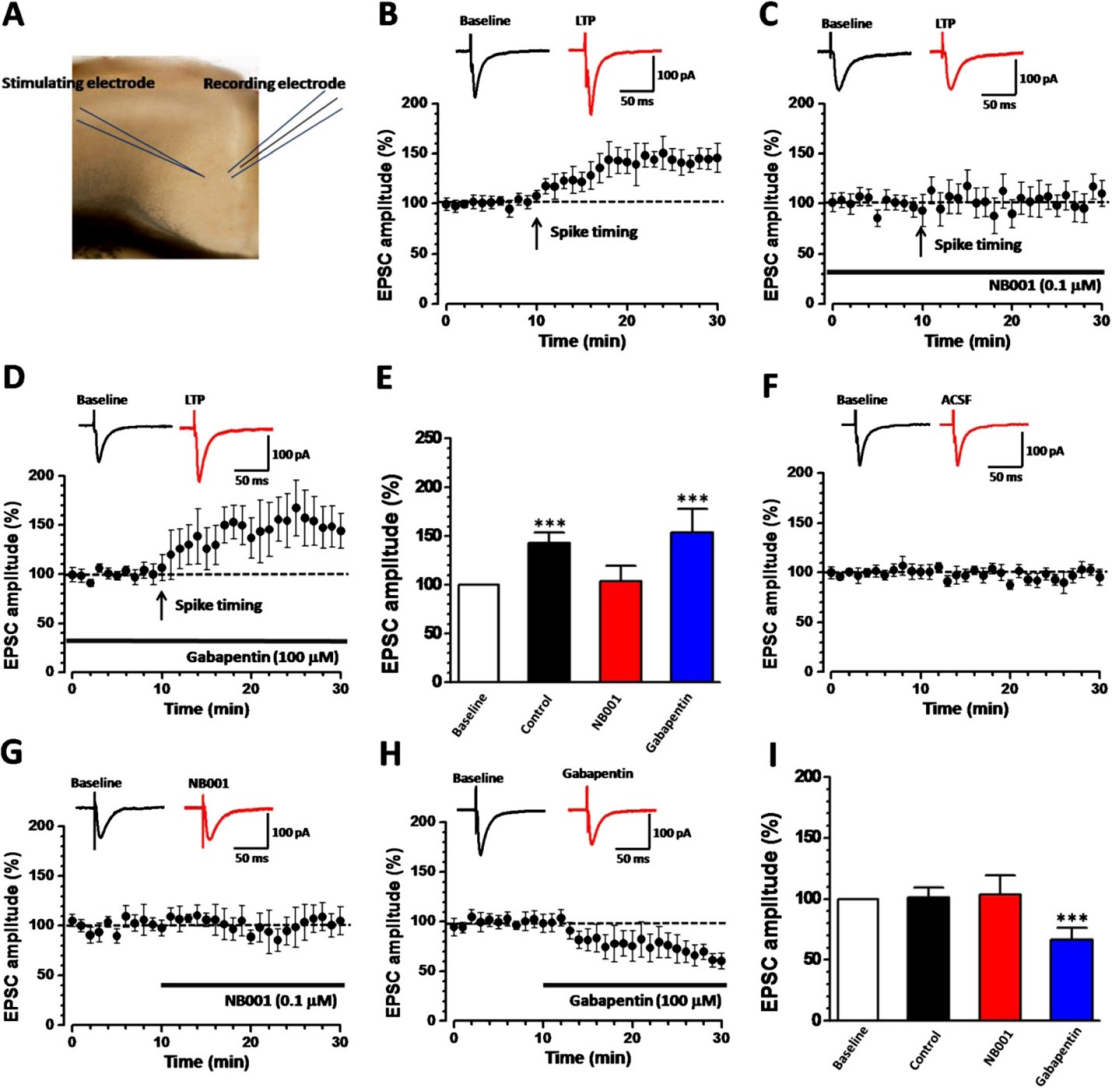
**Effect of gabapentin and NB001 on the LTP and basal synaptic transmission. (A)** Diagram of one ACC slice showing the placement of the whole cell patching system: stimulation electrode was placed on layer V and pyramidal cells in layer II/III were recorded. **(B)** The superimposed sample traces and the averaged results showed that spiking timing protocol could induce LTP lasted for 30 min in pyramidal neurons. **(C-D)** Spike timing protocol induced LTP was blocked in the presence of NB001 (0.1 μM, **C**) but not gabapentin (100 μM, **D**). **(E)** The summarized results of B-D were plotted. **(F)** Basal synaptic transmission was stable in control slice. **(G-H)** The basal synaptic transmission was not affected by NB001 **(G)** but reduced by gabapentin application **(H)**. **(I)** The summarized results of **F**-**H** were plotted.

To confirm the results from the multi-channel recording, the effects of NB001 and gabapentin were also tested by using whole-cell patch-clamp recording method. One site in deep layer V was stimulated and the evoked excitatory postsynaptic currents (eEPSCs) in superficial layers (II/III) were recorded (Figure 6A). Ten min after a stable baseline recording, spike timing protocol [14] were applied and an obvious LTP of the synaptic responses were induced. The averaged amplitude of EPSCs increased to 143.4 ± 10.9% of the baseline at 30 min after induction (*P <* 0.001, paired t-test; n = 6 neurons/6 slices, Figure 6B, E). In accordance with the field recording results, incubation with NB001 (0.1 μM) blocked the potentiation (102.2 ± 13.3% of the baseline at 30 min after induction, *P* = 0.143, n = 6 neurons/6 slices, Figure 6C, E). However, bath application of gabapentin (100 μM) did not block the spike timing induced potentiation (155.5 ± 24.2% of baseline at 30 min after induction, *P <* 0.001, n = 6 neurons/6 slices, paired t-test, Figure 6D, E).

### Effects on basal synaptic transmission

**Figure 7:**
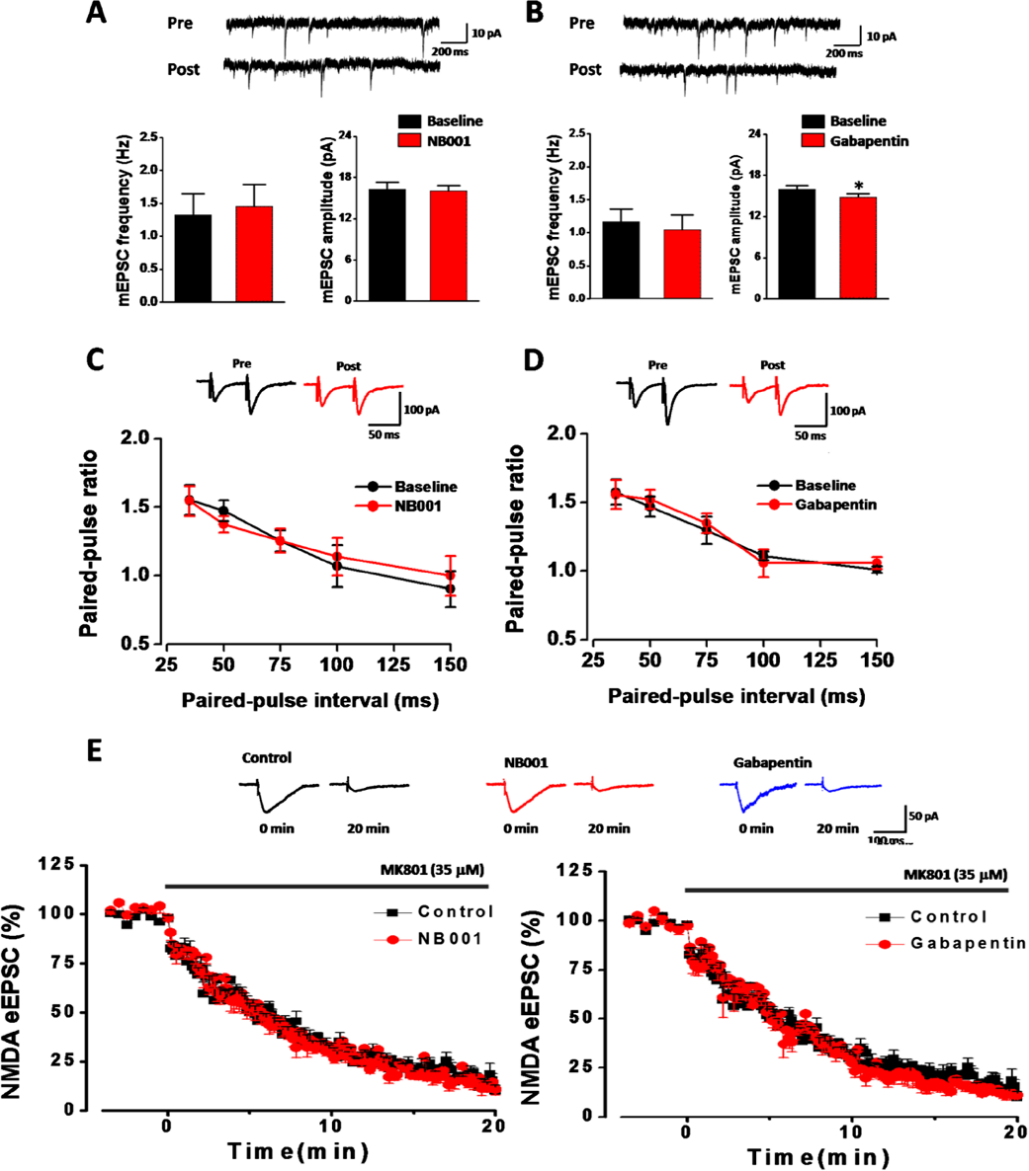
**NB001 and gabapentin did not change the presynaptic release of glutamate. (A)** Superimposed samples and summarized results showed that the baseline (pre) of the frequency and amplitude of mEPSCs was not changed by NB001 (0.1 μM, post) application. **(B)** Gabapentin (100 μM) only decreased the amplitude but not the frequency of the mEPSC. (C-D) NB001 **(C)** and gabapentin **(D)** had no effect on the paired pulse ratio recorded with intervals of 35, 50, 75, 100 and 150 ms. **(E)** Faster MK-801 blockade of NMDA EPSCs. Representative traces showed NMDA EPSCs from 0 to 20 min in the presence of MK-801 (35 μM) with or without NB001 or gabapentin application. NB001 and gabapentin has no effect on the plotted time course of MK-801 blockade of NMDA EPSCs.

We then tested the effects of NB001 and gabapentin on the basal synaptic transmission. Similar to the field recording results, we found that NB001 did not affect the amplitude of the evoked EPSCs (106.8 ± 15.5% of the baseline 20 min after bath application, *P* = 0.359, n = 6 neurons/6 slices, paired t-test; Figure 6G, I). However, gabapentin significantly reduced the amplitude of the eEPSCs (68.4 ± 9.9% of the baseline 20 min after bath application, *P* < 0.001, n = 6 neurons/6 slices; paired t-test; Figure 6H, I). To determine whether the synaptic changes observed with NB001 and gabapentin are due to presynaptic or postsynaptic mechanisms, AMPA receptor-mediated miniature EPSCs (mEPSCs), paired-pulse ratio (PPR) and fast blockade of NMDA receptor-mediated eEPSCs were tested [19, 20]. According to the quantal analysis, changes in the amplitude of mEPSCs are related to the postsynaptic alteration, while changes in the frequency typically reflect the alteration in presynaptic glutamate release [16, 37, 38]. Next we tested whether bath application of NB001 and gabapentin affect the amplitude or the frequency. We found that NB001 had no effect on both the frequency (Baseline: 1.2 ± 0.3 Hz, NB001: 1.3 ± 0.3 Hz, *P* = 0.135, paired t-test, n = 8 neurons/8 slices) and amplitude (Baseline: 15.5 ± 0.8 pA, NB001: 15.8 ± 0.7 pA, *P* = 0.673, paired t-test, n = 8 neurons/8 slices) of the mEPSCs (Figure 7A). However, gabapentin application caused a significant decrease in the amplitude of mEPSCs (Baseline: 15.8 ± 0.3pA, gabapentin: 14.8 ± 0.4 pA, *P* = 0.01, paired t-test, n = 7 neurons/7 slices), without affecting the frequency (Baseline: 1.1 ± 0.2 Hz, gabapentin: 1.1 ± 0.1 Hz, n = 7 neurons/7 slices, *P* = 0.99, paired t-test; Figure 7B). These results indicate that gabapentin may decrease the basal transmission through postsynaptic mechanisms.

Similar with the mEPSCs frequency analyses, paired-pulse ratio is a simple form of presynaptic plasticity, in which the response to the second stimulus is enhanced as a result of residual calcium in the presynaptic terminal after the first stimulus [38, 39]. Thus, to further determine the possible presynaptic change after NB001 and gabapentin application, we examined the PPR at different stimulus intervals of 35, 50, 75, 100 and 150 ms. We found that neither NB001 nor gabapentin changed the PPR in the ACC neurons (NB001: *F*_*(1, 70)*_ = 0.012, *P =* 0.913; n = 8 neurons/8 slices; Gabapentin:*F*_*(1, 60)*_ = 0.006, *P =* 0.937, n = 7 neurons/7 slices, Two-way ANOVA; Figure 7C-D). These results further indicate that the effect of NB001 and gabapentin are not associated with presynaptic release, although they have different effect on the basal synaptic transmission.

It has been widely reported that the blocking rate of NMDA receptor-mediated synaptic current by MK-801, a selective and non-competitive NMDA receptor antagonist, is correlated with glutamate release probability [40, 41]. Thus, testing the blocking rate of NMDA current could reflect whether the release probability was changed [19]. In slices with or without NB001 or gabapentin, we found that MK-801 could progressively block NMDA EPSCs and completely inhibited the current in 20 min (Figure 7E). By analyzing the time required for peak amplitude of NMDA EPSC to 50% decay of initial value in MK-801, we found no significant differences of the blocking rates among NB001-, gabapentin-treated and control groups (control: 5.85 ± 0.41 min; NB001: 5.78 ± 0.55 min; gabapentin: 5.98 ± 0.55 min. NB001 vs. control, *P* = 0.998; gabapentin vs. control, *P* = 0.694, One-way ANOVA, both from 7 neurons/7 slices). These results indicate that neither NB001 nor gabapentin affect the probability of presynaptic neurotransmitter release. Taken together, all these results suggest that NB001 has no effect on the basal synaptic transmission, whereas gabapentin may decrease the synaptic transmission through inhibiting the postsynaptic elements, instead of inhibiting the presynaptic glutamate release in the ACC.

## Discussion

In the present study, by using multi-channel recording system and whole-cell patch-clamp recording methods, we tested the effect of an adenylyl cyclase1 (AC1) inhibitor NB001 on the cortical L-LTP. We provide the first evidence that AC1 is required for the L-LTP in the adult mouse ACC. We also examined the effects of gabapentin in parallel for comparison. We found that NB001 at a significant low dosage totally blocked the induction of L-LTP and the recruitment of cortical circuit. By contrast, NB001 does not affect basal excitatory transmission. To our surprise, gabapentin has no effect on the L-LTP even at a much higher dosage. Gabapentin reduces basal synaptic transmission. These results suggest that NB001 and gabapentin produce analgesic effects in animal models of chronic pain by different central mechanisms.

AC1 and 8 are two key enzymes that produce second messenger cAMP following activation of intracellular calcium signaling pathway. In the hippocampus, calcium-stimulated AC activity is critical for long-term memory and L-LTP [42]. Both AC and AC8 activity are required for hippocampal L-LTP, and single deletion of AC1 or AC8 did not affect L-LTP in the CA1 region of the hippocampus [42]. Consistently, bath application of NB001 did not affect hippocampal E-LTP [21]. However, in the ACC, our previous works [21] and the present study showed that genetic or pharmacological inhibition of AC1 alone blocked both the E-LTP and L-LTP. Since LTP in the ACC is required for long-term plasticity that contributes to the development of chronic pain [4, 5], inhibiting AC1 activity by NB001 could be used to inhibit injury-related cortical plasticity. Because hippocampal LTP can be compensated by AC8 and other ACs, cognitive and emotional functions are unlikely affected [21]. Furthermore, AC1 inhibitor is highly selective for activity-dependent plasticity. Basal synaptic transmission is not affected by the same inhibitor, supporting the selectivity of the compound. Our results show that the popular analgesic drug gabapentin did affect normal synaptic transmission in the ACC. Furthermore, gabapentin failed to block ACC L-LTP. This explains why gabapentin has low clinical efficacy and high doses are needed to produce analgesic effects in neuropathic pain conditions. Moreover, the inhibitory effect on excitatory transmission is likely to be non-selective. It is consistent with clinical observations that gabapentin causes central side effects.

Our present studies confirm recent observation that L-LTP induction triggered the recruitment of cortical circuits (see [11]). Furthermore, we provide strong evidence that AC1 activity is required for the recruitment of silent responses during L-LTP. We propose that such recruitment may be caused by activation of ‘silent’ synapses [43–45]. The cortical recruitment is unlikely caused by simple enhancement of synaptic transmission. Our findings indicate that there are at least two basic mechanisms that may contribute to cortical potentiation at the circuit level. The first one is typical LTP, potentiation of synaptic responses by postsynaptic receptor modifications (see [5]). Another possibility is to recruit postsynaptic AMPA receptors into ‘silent’ synapses. In the ACC neurons, both forms of potentiation/recruitment require the activity of calcium stimulated AC1. However, up to now, it has been reported that silent synapses are mostly found in animals within two postnatal weeks [43, 46]. However, morphological studies show that certain matured synapses contain only NMDA receptors in adult hippocampus [47, 48]. Future studies are clearly needed to investigate this in adult ACC synapses.

**Figure 8:**
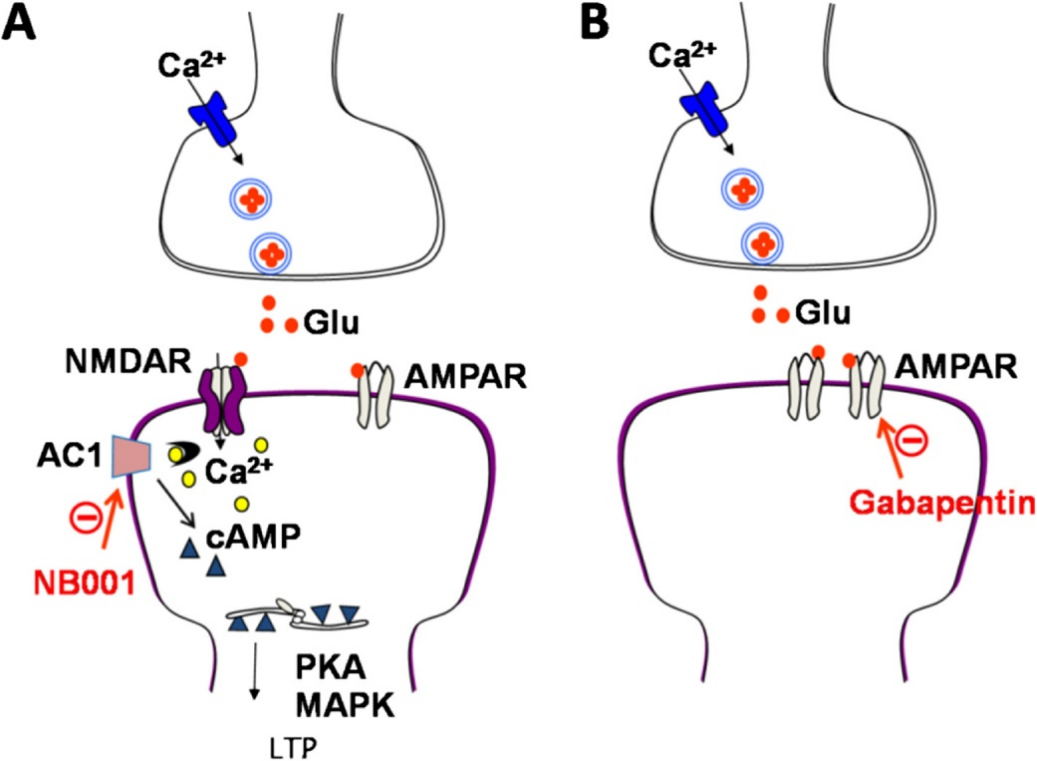
**A Model explains synaptic mechanisms of NB001 and gabapentin in the ACC. (A)** A synaptic model showing AC1 acts downstream from the glutamate NMDA receptors and activated in a calcium-dependent manner. NB001 inhibited the AC1 as a drug target for blocking the LTP in the ACC. **(B)** Gabapentin may have inhibitory effect on the AMPAR mediated synaptic transmission.

Cumulative evidence from both human and animal studies demonstrates that the cellular and molecular mechanism of acute and chronic pain is different [4, 5]. Physiological pain is probably mediated by the changed intensity of basal sensory synaptic transmission, without long term synaptic plasticity and new synthesis of activity-dependent signaling proteins. By contrast, pathological pain is probably involved with the long term synaptic potentiation, which is triggered by transcriptional and translational events, and might also result in structural changes and synthesis of new functional synapses [3, 6, 7, 49]. We found that NB001 only inhibit the LTP and channel recruitment without affecting the basal NMDA receptor or AMPA receptor mediated synaptic transmission [21]. In contrast, gabapentin, even in high concentration (100 μM), cannot block the induction of LTP and synaptic propagation. The results thus suggest NB001 and gabapentin may cause analgesic effect by different mechanism: NB001 mainly prevents the development of “pathological LTP”, while gabapentin causes general inhibition of the basal synaptic transmission (Figure 8). Based on these observations, we believe that NB001 should be a better candidate for analgesic drugs comparing with gabapentin. Furthermore, in addition being more effective, NB001 should cause lesser side effects than gabapentin. Central side effects such as dizziness, drowsiness, depression and even suicide, after high dose application of gabapentin has been widely reported [50, 51]. Our present results provide the first study to show the effect of AC1 inhibitor NB001 on the late-phase LTP and spatial propagation in the ACC of adult mice. NB001, in comparison with the widely used analgesic gabapentin, will be more selective for pathological pain treatment.

## Methods

### Animals

Adult (8–10 weeks) male C57BL/6 mice were used. All animals were housed under a 12 h light/dark cycle with food and water provided *ad libitum*. The Animal Care and Use Committee of the Xi’an Jiaotong University approved all mouse protocols.

### Brain slice preparation

Mice were anesthetized with ether and decapitated. The whole brain was rapidly removed and transferred to ice cold oxygenated (95% and 5%) artificial cerebrospinal fluid (ACSF) containing (in mM): 124NaCl, 25 NaHCO_3_, 2.5 KCl, 1 KH_2_PO_4_, 2 CaCl_2_, 2 MgSO_4_ and10 glucose, pH 7.4. After cooling for about 1–2 min, appropriate portions of the brain were then trimmed and the remaining brain block was glued onto the stage of a vibrating tissue slicer (Leica VT1200S). Three coronal brain slices (300 μm), after the corpus callosum meets and contains ACC, were cut and transferred to a chamber with oxygenated ACSF at room temperature for at least 1.5 h [52].

### Preparation of the multi-electrode array probe

The procedures for preparation of the MED64 probe were similar to those described previously [11]. The MED64 probe (MED-P515A, 8 × 8 array, inter-polar distance 150 μm) was perfused with ACSF at 28–30°C with the aid of a peristaltic pump (Minipuls 3, Gilson) during the whole experimental period of electrophysiological recording. Before use, the surface of the MED64 probe was treated with 0.1% polyethyleneimine (Sigma, St. Louis) in 25 mM borate buffer (pH 8.4) overnight at room temperature. In addition, the probe surface was rinsed 3–5 times with sterile distilled water before immediate use in each experiment.

### Field potential recording in the ACC slices

After incubation, one slice was transferred to the recording chamber and suffused with ACSF at a 2 ml/min flow rate. The slices were positioned on the MED64 probe in such a way that the whole array of the electrodes can cover the different layers of the ACC, with middle part of the probe close to the central point of the ACC. One of the channels located in the layer V of the ACC, from which the best synaptic responses can be induced in the surrounding recording channels, was then chosen as the stimulation site. Channels in which field potentials can be induced were considered as active. A microphotograph of one ACC slice positioned on the MED64 probe was shown in Figure 1A-B. After the baseline responses were stabilized for at least 1 h, a TBS protocol (10 bursts at 5 Hz, 4 pulses at 100 Hz for each burst) was given 5 times (10 s interval) at the same stimulation site to induce L-LTP.

### Whole-cell patch-clamp recording

For whole cell patch clamp recording, slices were transferred to a submerged chamber and superfused (2 ml/min) with oxygenated ACSF at 28–30°C. Experiments were performed in a recording chamber on the stage of a BX51W1 (Olympus) microscope equipped with infrared DIC optics for visualization. Excitatory postsynaptic currents (EPSCs) were recorded from superficial layers neurons with an Axon 200B amplifier (Axon Instruments). Patch pipettes with resistances of 3–5 MΩ were filled with the following solution (in mM): 120 K-gluconate, 5 NaCl, 1 MgCl_2_, 0.2 EGTA, 10 HEPES, 2 Mg-ATP, 0.1 Na_3_-GTP and 10 phosphocreatine disodium (adjusted to pH 7.2 with KOH). The local stimulations were delivered by a bipolar tungsten stimulating electrode placed in deep layer (layer V/VI). AMPA receptor–mediated EPSCs were induced by repetitive stimulations at 0.05 Hz, and neurons were voltage-clamped at -60 mV. LTP was induced by spike timing protocol (three presynaptic stimuli at 30 Hz, which caused three EPSPs, were paired with three postsynaptic action potentials (APs), and did 15 times with an interval of 5 s. Presynaptic stimulus was delivered 10 ms before postsynaptic AP). For miniature EPSC (mEPSC) recording, 0.5 μM tetrodotoxin was added to the perfusion solution. Picrotoxin (100 μM) was always present to block GABA_A_ (γ-aminobutyric acid type A) receptor–mediated inhibitory synaptic currents both in AMPA receptor-mediated EPSCs and mEPSC. NMDA EPSCs were recorded for 20 min in MK-801 (35 μM) with 0.1 Hz stimulation [14].

### Drugs

The chemicals and drugs used in this study were as follows: gabapentin, MK-801, picrotoxin and tetrodotoxin were purchased from Sigma (St. Louis). Drugs were prepared as stock solutions for frozen aliquots at -20°C. All these drugs were diluted from the stock solutions to the final desired concentration in ACSF before used.

### Data analysis

Whole-cell patch-clamp data were collected and analyzed with Clampex 10.3 and Clampfit 10.2 software (Axon Instruments). MED64 Mobius was used for data acquisition and analysis. The percentages of the fEPSP slopes were normalized by the averaged value of the baseline. We defined LTP in a channel if the response was increased by at least 15% of baseline during this period. Results are expressed as mean ± SEM. The student’s *t*-test, One-way ANOVA and two-way ANOVA were used for Statistical comparisons. The level of significance was set at *P* < 0.05.

## Abbreviations

AC: Adenylyl cyclase
ACC: Anterior cingulate cortex
ACSF: Artificial cerebrospinal fluid
AMPAR: α-amino-3-hydroxy-5-methyl-4-isoxazole-propionic acid receptor
APs: Action potentials
eEPSC: Evoked excitatory postsynaptic current
E-LTP: Early-phase long term potentiation
EPSC: Field excitatory postsynaptic current
fEPSP: Field excitatory postsynaptic potentials
GABA_A_: γ-aminobutyric acid type A
L-LTP: Late-phase long term potentiation
mEPSC: Miniature excitatory post-synaptic current
NMDAR: N-methyl-D-aspartic acid receptor
PPF: Paired-pulse facilitation
TBS: Theta burst stimulation.
